# *Mycobacterium avium* Subspecies *paratuberculosis*: Human Exposure through Environmental and Domestic Aerosols

**DOI:** 10.3390/pathogens3030577

**Published:** 2014-07-16

**Authors:** Glenn Rhodes, Hollian Richardson, John Hermon-Taylor, Andrew Weightman, Andrew Higham, Roger Pickup

**Affiliations:** 1 Centre for Ecology and Hydrology, Lake Ecosystems Group, Lancaster Environment Centre, Library Avenue, Bailrigg, Lancaster LA1 4AP, UK; E-Mail: Glenn@ceh.ac.uk; 2 Faculty of Health and Medicine, Division of Biomedical and Life Sciences, Lancaster University, Lancaster LA1 4YQ, UK; E-Mail: h.richardson2@lancaster.ac.uk; 3 Division of Diabetes and Nutritional Sciences, Franklin-Wilkins Building, King’s College London, 150 Stamford Street, London SE1 9NH, United Kingdom; E-Mail: j.hermon@kcl.ac.uk; 4 Cardiff School of Biosciences, Main Building, Museum Avenue, Cardiff CF10 3AT, UK; E-Mail: a.weightman@cardiff.ac.uk; 5 Royal Lancaster Infirmary, Ashton Road, Lancaster, Lancashire LA1 4RP, UK; E-Mail: Andrew.Higham@mbht.nhs.uk

**Keywords:** *Mycobacterium avium* subspecies *paratuberculosis*, Crohn’s disease, aerosols, rivers, domestic showers, exposure

## Abstract

*Mycobacterium avium* subspecies *paratuberculosis* (*Map*) causes Johne’s disease in animals and is significantly associated with Crohn’s disease (CD) in humans. Our previous studies have shown *Map* to be present in U.K. rivers due to land deposition from chronic livestock infection and runoff driven by rainfall. The epidemiology of CD in Cardiff showed a significant association with the River Taff, in which *Map* can be detected on a regular basis. We have previously hypothesized that aerosols from the river might influence the epidemiology of CD. In this preliminary study, we detected *Map* by quantitative PCR in one of five aerosol samples collected above the River Taff. In addition, we examined domestic showers from different regions in the U.K. and detected *Map* in three out of 30 independent samples. In detecting *Map* in river aerosols and those from domestic showers, this is the first study to provide evidence that aerosols are an exposure route for *Map* to humans and may play a role in the epidemiology of CD.

## 1. Introduction

*Mycobacterium avium* subspecies *paratuberculosis* (*Map*) is a member of the *Mycobacterium avium* complex [1,2]. It has the specific ability to cause chronic inflammation of the intestine, or Johne’s disease (JD) [3,4,5], which can affect many animal species, including primates [6,7]. This enteric pathogen is significantly associated with chronic inflammation of the intestine of the Crohn’s disease (CD) type in humans [8,9,10].

Subclinical infection is widespread in domestic livestock, especially cattle, sheep and goats [3]. Infection and disease has now spread worldwide [11,12], with Europe and North America being particularly affected [3,13]. It is estimated that the herd prevalence for JD in cattle in the USA is 68% [14] and 32% in U.K. [3,15,16]. Both clinically and sub-clinically infected animals can shed *Map* in variable numbers on to pasture in their faeces, depending on the animal, the pathogen strain and the disease characteristics [6]. The organism can survive for many months in agricultural slurry and in the wider environment [17,18,19,20].

Rain falling onto pastures contaminated with *Map* washes it into surface waters and into rivers [19,20]. Previously, we showed that *Map* was present in 32% of samples taken from the River Taff (Cardiff, South Wales, UK) and its presence was almost predictable from rain fall patterns and river flow [19], whereas its frequency in samples from the River Tywi was 69% and its presence predictable [20]. Furthermore, deposition and transport from the catchment was extensive in that *Map* was maintained in the river for several weeks at a time and was a consequence of the endemic presence of *Map* in cattle in the Taff catchment [19]. Pickup and co-workers [20] modelled the main human exposure routes of *Map* and suggested that although driven by shedding from clinically and sub-clinically infected animals, the presence and distribution of *Map* in the environment may also be influenced by other factors, such as slurrying and soil redistribution from water treatment that recycles *Map* back from the catchment to the river [19,20]. Water from rivers or reservoirs is used for abstraction and public supply. Inevitably *Map* has been detected in the drinking water supply systems [20,21,22,23,24,25,26]. Furthermore, mycobacteria, including *M. avium* subspecies *avium*, have been found in domestic showers [27,28,29].

**Figure 1:**
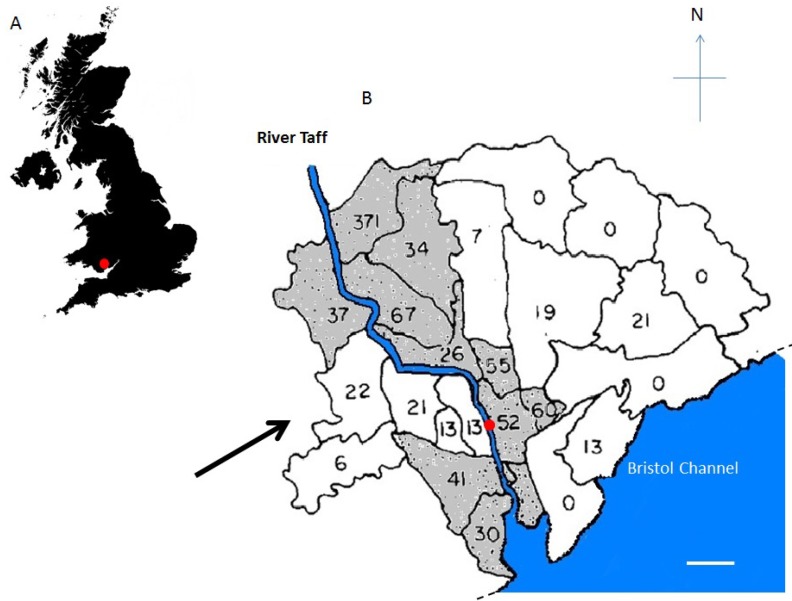
Relationship between disease clusters and prevailing wind in Cardiff, Wales, United Kingdom. (**A**) Location of the city of Cardiff within the UK (red dot); (**B**) distribution of the 11 electoral wards (shown with black boundaries) in the city of Cardiff that were shown previously (53, 54) to have a highly significant (*p* < 0.001) increase in the incidence of Crohn’s disease. The wards with the high incidence of Crohn’s disease are seen to lie along the River Taff (blue), which flows into the Bristol Channel (blue), with the exception of a gap in the centre stretch of the windward right bank of the river (facing downstream). This gap directly faces a valley between hills to the north and south, which is open to the prevailing southwesterly winds (black arrow; with permission from [19]). The dashed line represents the continuation of the coast. The white bar represents 3 km. The red dot represents the sampling site.

Cardiff has one of the highest incidences of CD in the U.K., with a corrected incidence for the decade (1996–2005) of 6.6 per 10^5^ population per year (with a 95% confidence interval of 58–76) with a concomitant increase in incidence in children under 16 [30,31]. This compares with 5.6 per 10^5^ population per year (with a 95% confidence interval of 44–68) for the period 1991–1995 [32], 5.9/10^5^/year for 1986–1990 [33] and 5.0/10^5^/year over the period from 1976 to 1980 [34]. The epidemiological study carried out by Mayberry and Hitchens in the late 1970s [35] examined the incidence and geographical distribution of Crohn’s disease and ulcerative colitis in 25 electoral wards in the city of Cardiff. They found a statistically highly significant (*p* < 0.001) increase in the incidence of Crohn’s disease, but not of ulcerative colitis, in 11 of the city wards. Although eight of these high-incidence wards bordered the River Taff, their apparent geographical relationship with the river was not statistically significant [35,36]. Figure 1B shows the city of Cardiff with an indication of the direction of the prevailing south westerly winds [19]. The distribution of the wards with a high incidence of Crohn’s disease throughout the city is shown in Figure 1B [19]. The topography of the approaches to the southwest aspect of the river is characterized by hills to the north and south. The valley in between, open to the prevailing winds, is directly opposite the gap in the centre of the high-incidence wards on the windward bank of the river. On the leeward side, three additional high-incidence wards lie immediately adjacent to those bordering the river to the northeast. Previously, we suggested that this is the direction in which aerosols containing *Map* would be carried on the prevailing south westerly winds and proposed that, through aerosolisation of *Map*, this exposure route is an explanation for the observed disease clusters around the River Taff [19].

**Figure 2:**
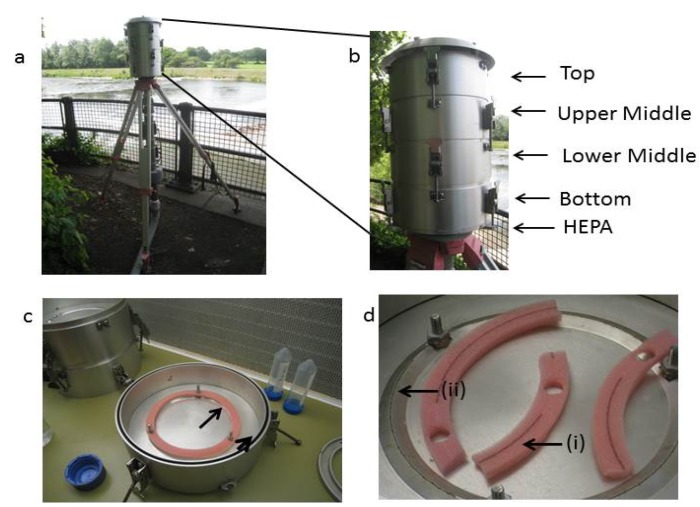
Aerosol sampling using a high volume impaction system. (**A**) The system in place connected to a high volume vacuum pump (not shown); (**B**) the sampler showing the position of the four foam collection substrates; (**C**) the foam placed in position over one of the slit nozzle cascade impactors (see arrow); (**D**) the foam removed from the slit nozzle cascade impactors (ii) showing collected particles from the sampler (i).

In the present study, we tested whether we could detect *Map* in aerosols originating from the River Taff, using a high volume impaction sampler (HVIS) designed for collecting particulate matter (PM 2.5–10; Figure 2) [35,36,37]. We also investigated domestic showers to find out whether they could be a source of exposure; thus seeking to extend the diverse routes for human exposure proposed by Pickup *et al*. [20].

## 2. Results

### 2.1. Aerosols

#### 2.1.1. The Efficiency of Recovery of Bacteria from the Foam Collection Substrate

This is the first study to make use of the HVIS for microbiological analysis. The foam collection substrate, originally designed for particulate matter (PM10) capture, was tested for ease of recovering culturable bacteria, as it was anticipated that there would be some cell loss due to adsorption and derived from the clumping associated with mycobacteria. In separate experiments, foams were infused with *E. coli* JM101 and *M. immunogenum* over a serial dilution range delivering cell numbers from 10–10^7^ CFU mL^−1^ for *E. coli* and 10–10^5^ CFU mL^−1^ for *M. immunogenum*. For *E. coli*, the efficiency of recovery decreased with decreasing infusion concentration from approximately 200% (for 10^6^/10^7^ CFU mL^−1^ infusions, possibly due to cell multiplication at high cell density) to 69%–43% for an infused cell concentration in the range of 10–10^5^, respectively. No CFU were recovered when the foam was seeded with 10 CFU mL^−1^. *M. immunogenum* was infused in the range 10–10^5^ CFU mL^−1^ with CFU only being recovered from the 10^5^ cell infusion at an efficiency of 0.05%. The detection of *M. immunogenum* by qPCR demonstrated variable recoveries of between 0.08% and 3.05% for the cell equivalent (CE) infusion range 10–10^7^, respectively, indicating better recovery at low cell density. No CE were detected by qPCR when initially infused below concentrations of 10^1^ CE mL^−1^. Overall results indicated that bacteria could be recovered from the foam collection substrate, originally designed for PM10 collection, but the efficiency of recovery is likely dependent on the concentration and cell type.

#### 2.1.2. Analysis of River Taff Foam Collection Substrates

##### 2.1.2.1. Culture

River aerosols were obtained on five occasions, with each filtering a total of 198 m^3^ of air per sampling session. Both heterotrophic plate counts and direct microscope counts were carried out on the bacteria recovered from the foam collection substrates located at the top, middle and bottom of the HVIS (Table 1; Figure 2).

**Table 1:** Assessment of the bacterial load of aerosols from the River Taff by epifluorescence microscopy and the culture of general heterotrophic bacteria. (Samples were collected between November, 2010, and September, 2011; ND, not determined).

Date (MM/DD/YY)	Filter Level	Culturable Counts (R2A Agar) Mean CFUs m^−3 ^ (Standard Deviation)	Direct Counts (DAPI) Mean Cells m^−3^ (Standard Deviation)
11.09.10	Top	1.11 × 10^4^ (±3.21 × 10^3^)	1.89 × 10^5^ (±9.62 × 10^4^)
	Upper Middle	6.01 × 10^4^ (±1.17 × 10^4^)	1.60 × 10^5^ (±6.27 × 10^4^)
	Lower Middle	ND	ND
	Bottom	1.19 × 10^2^ (±1.06 × 10^2^)	ND
05.24.11	Top	1.58 × 10^4^ (±8.17 × 10^32^)	2.58 × 10^5^ (±1.47 × 10^5^)
	Middle	ND	ND
	Bottom	ND	ND
06.15.11	Top	5.65 × 10^4^ (±2.56 × 10^4^)	ND
	Middle	ND	ND
	Bottom	ND	ND
08.17.11	Top	5.86 × 10^4^ (±4.23 × 10^3^)	5.20 × 10^7^ (±2.46 × 10^7^)
	Upper middle	1.27 × 10^5^ (±2.45 × 10^4^)	ND
	Lower middle	2.12 × 10^2^ (±9.68 × 10^1^)	ND
	Bottom	0	ND
09.21.11	Top	3.50 × 10^4^ (±2.19 × 10^4^)	2.75 × 10^5^ (±1.64 × 10^5^)
	Upper middle	2.26 × 10^3^ (±1.73 × 10^2^)	3.91 × 10^4^ (±1.16 × 10^4^)
	Lower middle	3.90 × 10^1^ (±5.08 × 10°)	ND
	Bottom	0	ND

The foams collected bacteria in decreasing numbers from “top” to “bottom” in the mean range 6.01 × 10^4^ to 3.90 × 10^1^ CFU m^−3^ for culturable bacteria and in the mean range 5.2 × 10^7^ to 3.91 × 10^4^ CFU m^−3^ for direct counts. There was no significant difference between the culturable numbers collected on the top and upper middle foams (mean 4.7 × 10^5^ ± 7.2 × 10^5^ CFU m^−3^), but the difference in numbers between the top and lower middle and bottom were close to being significantly different (*p* = 0.06). Direct counts by microscopy for top and upper middle foams had a mean 8.8 × 10^7^ ± 2.1 × 10^8^ cells m^3^, with no significant difference between top and upper middle foams. Therefore, the HVIS collected the majority of bacteria from the air passing over the river in the top and upper middle collection substrates.

##### 2.1.2.2. Molecular Analyses

16S *rrn* gene amplification was successful from all top and upper middle section foams throughout the sampling period. The bottom sections were all negative. PCR detection was not observed in samples where general heterotrophs numbered <10^3^ CFU m^−3^ (Table 2).

**Table 2:** PCR detection of eubacteria (16S *rrn* gene) *Mycobacterium* spp. (gMyc) and Map (IS*900* and F57) in aerosol collection foams collected from November, 2010, to September, 2011 (CE, cell equivalents).

Date (MM/DD/YY)	Filter Level	Eubacterial 16S *rrn* Gene	*Mycobacterium* spp. 16-23S rRNA ITS (gMyc) (CE m^−3^)	Map IS*900* (CE m^−3^)	Map F57 (CE m^−3^)
11.09.10	Top	+	0	0	0
	Upper Middle	+	0	1–10	0
	Lower middle	-	0	0	0
	Bottom	-	0	0	0
05.24.11	Top	+	0	0	0
06.15.11	Top	+	0	0	0
08.17.11	Top	+	1–10	0	0
	Upper middle	+	0	0	0
	Lower middle	-	0	0	0
	Bottom	-	0	0	0
09.21.11	Top	+	0	0	0
	Upper middle	+	0	0	0
	Lower middle	-	0	0	0
	Bottom	-	0	0	0

*Mycobacterial* spp. were detected using the gMyc genus-specific assay in the foam on one occasion (08.17.11; Table 2). *Map* was detected by PCR (IS*900* only) on a different occasion (11.09.10) and was found in the upper middle filter. *Map* was not detected by the confirmatory, but less sensitive, F57 PCR on any occasion. In summary, bacteria were detected on collection foams by culture and direct counts; the extracted DNA was of sufficient quality for PCR, and *Map* was detected in one sample by IS*900* PCR.

### 2.2. Showers

Samples from the biofilms in showers tubes and heads were obtained from 23 homes across four counties in the U.K. ((Cumbria, Lancashire, Merseyside and West Sussex; 30 samples in total); Table 3).

**Table 3:** Detection of *Mycobacterium* spp. and *Mycobacterium avium* subspecies *paratuberculosis* in shower heads and tubes by culture and qPCR (ND, not determined; H represents a shower head being sampled from the same shower unit; a, b represent different showers from the same house; and numbers represent different locations). MGIT, mycobacterial growth indicator tubes.

Sample Location/Number	Microscopy and Culture		qPCR		
	Direct Counts and Standard Deviation. (Cells L^−1^)	Mycobacterial Culture (MGIT)	*Mycobacterium* spp. (CE L^−1^)	*Map* IS*900* (CE L^−1^)	*Map* F57 (CE L^−1^)
Cumbria-1	ND	ND	10^4^–10^5^	0	0
Cumbria-2H	ND	-	10^6^–10^7^	0	0
Cumbria-2	ND	-	10^6^–10^7^	0	0
Cumbria-3	ND	-	10^3^–10^4^	0	0
Cumbria-4	ND	-	10^2^–10^3^	0	0
Cumbria-5	ND	ND	0	0	0
Cumbria-6	ND	ND	0	0	0
Lancashire-1	ND	ND	10^3^–10^4^	0	0
**Lancashire-2**	**ND**	**ND**	**10^7^–10^8^**	**1–10**	**0**
Lancashire-3	ND	-	10^7^–10^8^	0	0
Lancashire-4	7.95 × 10^8^ (±4.02 × 10^8^)	-	10^5^–10^6^	0	0
Lancashire-5	ND	-	10^6^–10^7^	0	0
Lancashire-6	ND	ND	10^8^–10^9^	0	0
Lancashire-7	ND	+	10^6^–10^7^	0	0
Lancashire-8	ND	-	10^3^–10^4^	0	0
Lancashire-9	1.91 × 10^9^ (±6.07 × 10^8^)	-	10^6^–10^7^	0	0
Lancashire-10	1.63 × 10^9^ (±5.05 × 10^8^)	+	10^7^–10^8^	0	0
**Merseyside -1**	**ND**	**-**	**10^8^–10^9^**	**10^3^–10^4^**	**10^1^–10^2^**
**Merseyside-2**	**ND**	**-**	**10^7^–10^8^**	**10^2^–10^3^**	**10^2^–10^3^**
Merseyside-3	ND	-	10^7^–10^8^	0	0
West Sussex-1aH	ND	ND	10^7^–10^8^	0	0
West Sussex-1a	ND	ND	10^6^–10^7^	0	0
West Sussex-1bH	ND	-	10^6^–10^7^	0	0
West Sussex-1b	ND	+	10^7^–10^8^	0	0
West Sussex-2a	1.64 × 10^9 ^(±7.39 × 10^8^)	-	10^9^–10^1^°	0	0
West Sussex-2b	2.25 × 10^9 ^(±1.02 × 10^9^)	-	10^4^–10^5^	0	0
West Sussex-3aH	ND	-	10^4^–10^5^	0	0
West Sussex-3a	ND	-	10^5^–10^6^	0	0
West Sussex-3b	ND	+	10^5^–10^6^	0	0
West Sussex-4H	ND	-	10^4^–10^5^	0	0

*Mycobacterium* spp. were detected by qPCR in 28 (93%) samples covering all geographical regions. Their numbers ranged from 10^2^ CE L^−1^ to 10^9^–10^10^ CE L^−1^ with >20 samples containing more than 10^2^–10^3^ CE L^−1^ mycobacteria. The presence of viable mycobacteria was also confirmed by culture (with confirmation of acid-fastness by Kinyoun staining) in four out of six samples tested (Table 3). *Map* was detected in three samples from Merseyside and Lancashire, two samples of which were also positive in a confirmatory F57 PCR. Furthermore, *Map* was only detected in samples carrying the higher 10^7^–10^8^ general mycobacterial load (Table 3).

### 2.3. Discussion

This study employed culture and higher sensitivity qPCR assays to assess two routes of human exposure to *Map* via aerosols, as proposed by Pickup *et al*. [19,20], namely, those from rivers and domestic showers.

#### 2.3.1. River Aerosols

Various types of collection devices are available, such as impingers with a low volume collection of 10–30 L min^−1^, although some have a capacity of approximately 1000 L min^−1^ [38,39]. Both dry and liquid collection systems have limitations, such as short collection times, impaction problems and low volume collection [38,40]. Our river sampling strategy for aerosols employed a high volume impaction system (HVIS; commonly referred to as the Cardiff Super-Sucker), which is normally used to study PM10 in urban and rural areas [37]. To our knowledge, there is no dedicated apparatus to facilitate the specific detection of aerosolized bacteria in such large volumes. As such, it was necessary to show the applicability of the HVIS system to the capture and recovery of bacteria in the present study. We demonstrated the use of the apparatus and showed that viable bacteria can be recovered from River Taff aerosols by culturing bacteria from 198 m^3^ air samples collected on each deployment. This recovery occurred despite the harsh physical and physiological conditions that the unit imposes on the bacteria and the non-standard collection foams used. The HVIS was placed near a weir, where the physical disturbance of the river would probably increase our chances of collecting aerosols.

*Map* was detected by IS*900* qPCR, on one out of five sampling occasions, albeit in the range of 1–10 CE m^3^, but not by the less sensitive F57 PCR, which is consistent with the estimated Map numbers detected. In a previous qualitative study (presence/absence PCR; [19]), Pickup *et al.* (2005) sampled the river Taff waters for one year and reported that 32% of river samples at a location 1 km upstream from the present site were positive for *Map* [19]*.* The 2005 study [19] was the result of a feasibility study comprising seven *ad hoc* sampling sessions, previously spread out over several months. *Map* was detected in only one of those seven samples. However, its detection led to the more intensive sampling regime that was later reported [19]. We subsequently showed that the *ad hoc* feasibly study returned a low *Map-*positive rate because the bacterium is transported in the river in pulses driven by rainfall and that there are periods where *Map* is not detected [19,20], and we sampled on all, but one, of those occasions. The conclusions of the present study mirror that of our previous studies [19,20,41] in that sampling and detection methods likely underestimate actual *Map* numbers. More specifically, previous experience from *ad hoc* sampling and *Map* screening followed by more intense and frequent assessments in 2005 suggest that if we were able to collect aerosol samples on a twice weekly basis at the present sampling site, then our model for the River Taff would suggest a higher detection of *Map* in aerosol samples [19]. This is our intention in future studies.

Both the ability of aerosol droplets to concentrate bacteria and the spread of mycobacteria in aerosols are well documented [23,42,43,44,45,46,47]. More specifically, *Map* has been detected in aerosols in cattle barns and within the farm environment [48,49,50,51] and has been identified as a possible route for infection for Johne’s disease in cattle [52]. Although limited in scope and resolution, our feasibility study is the first to show that *Map* is airborne in non-farm environments, namely in aerosols emanating from rivers, and its presence fits the criteria in our understanding of the *Map* loading of rivers and its transportation drivers from the catchment [19].

This study also gives credence to our suggestion that the epidemiology of Crohn’s patients in Cardiff (UK) is affected by aerosols, due to the significant association with the river and the prevailing wind direction [19]. The study by Mayberry and Hitchens [35] examined the distribution of Crohn’s disease patients in Cardiff based on age, sex, religion, smoking, drinking and drug taking with no statistical difference within and between these parameters [35]. They found, however, that CD patients appeared in highly significant clusters and that these were closely associated with the course of the River Taff with larger concentrations of CD nearer the source mouth (Tiger Bay). Mayberry and Hitchens [35] suggested that environmental factors associated with the river may contribute to the disease distribution. We suggest that, although more study is needed, aerosols might be the missing factor suggested by Mayberry and Hitchens [35,36,53,54] and ourselves [19]. Taken together with our present results and those that show that *Map* is significantly associated with Crohn’s disease [8,10], these data suggest that the pattern of clustering of Crohn’s disease in Cardiff may have been due to the long-term inhalation of *M. avium* subsp. *paratuberculosis* from the River Taff.

#### 2.3.2. Shower Heads

Furthermore, water is abstracted from rivers for domestic use. Given the widespread geographical distribution of *Map* [41] and its presence in water ways [19,20,24], it is not surprising that the delivery of domestic water supply may prove an issue. Mycobacteria have been found in drinking water [21,22,29], and pathogens, including mycobacteria, have been found in showerheads [27,29]. Furthermore, non-tuberculous mycobacteria (NTM) and other opportunistic human pathogens were enriched to high levels in many showerhead biofilms [27]. These authors concluded that showerheads may present a significant potential exposure to aerosolized microbes, including documented opportunistic pathogens. Our results support this with qPCR, showing 28 of 30 (93%) samples to be positive for *Mycobacterium* spp. in a range from 10^2^–10^10^ CE L^−1^. Additionally, we report for the first time the specific detection *Map* in shower tubes in numbers from 10^1^–10^4^ CE L^−1^ with two samples confirmed as *Map*-positive by both IS*900* and F57 PCR and one other unconfirmed *Map*-positive in which IS*900* was detected solely (1–10 CE 1^−1^). The presence in the shower tube will result in frequent sloughing from the surface of the biofilm, resulting in the appearance of *Mycobacterium* spp. and *Map* in shower water and the potential for subsequent inhalation via shower-generated aerosols as suggested by Feazel *et al*. [27].

## 3. Experimental Section

### 3.1. Bacterial Strains and Culture

*Escherichia coli* strain JM101 was maintained on nutrient agar and cultured overnight at 30 °C in nutrient broth (Oxoid, UK) with shaking at 180 rpm. General heterotrophs were cultured at 30 °C on solid R2A medium (Oxoid, UK) for 48 h. *Mycobacterium immunogenum* ATCC 700505^T^ was cultured at 30 °C for up to 1 week on Middlebrook 7H10 agar supplemented with OADC (10% v/v) and glycerol (5% v/v) (BD Biosciences, Oxford, UK) or in mycobacterial growth indicator tubes (MGITs), supplemented with OADC (10% v/v) [54]. *Mycobacterium* *avium* subsp. *paratuberculosis* K-10 (*Map*) and general mycobacteria from shower samples were cultured in the same way, but with the addition of polymyxin B sulphate (100 µg mL^−1^), cycloheximide (25 µg mL^−1^) and mycobactin J (2 µg mL^−1^); and increased incubation times of up to 6 months (*Map* culture only).

### 3.2. Sampling Sites

Aerosol sampling took place from the east bank on the River Taff at Blackweir, Bute Park Cardiff (GBOS ST170780), between November, 2010, and September, 2011. Shower samples were taken from a number of houses across England during the period 2011–2013.

### 3.3. Shower Sampling

Domestic shower samples were obtained by removal of the shower hose from the shower unit and shower head and emptied of standing water. Sterile glass beads (1 g; 5 mm in diameter) in 10 mL sterile 1 × PBS were poured into the pipe. The ends were sealed with alcohol-washed parafilm (Bemis Ltd, UK), and the pipe was manually shaken for 2 min to remove the internal biofilm. The contents were poured into a sterile McCartney bottle, sealed and put on ice prior to transport and processing: the culture of mycobacteria (Section 3.3.1), direct counts by microscopy (Section 3.5) and qPCR (Section 3.6 and Section 3.7). Counts obtained from 5-mL samples were converted to cells L^−1^ or CE L^−1^.

#### 3.3.1. Recovery of Mycobacteria from Shower Biofilms

For the recovery of mycobacteria from shower samples, 5 mL of the sample obtained above was centrifuged for 30 min at 4000× g. Samples were then decontaminated to favour the growth of robust mycobacteria, whilst eradicating other bacteria and fungi. Concentrated shower samples were re-suspended in 1 mL 0.7% hexadecylpyridinium chloride and incubated at room temperature for 1 h. This was followed by centrifugation for 30 min at 4000× g and re-suspension in 500 µL sterile PBS. Two hundred microlitres of this sample were then inoculated into MGITs and 100 µL onto 7H10 solid medium in triplicate, as described above. Mycobacterial growth either as colonies on solid media or from clumped cells in MGITs was confirmed by checking for both acid fastness by Kinyoun staining (as per the manufacturer’s instructions; Becton Dickinson Diagnostics, Oxford, UK) and DNA extraction followed by quantitative real-time PCR (qPCR) using the *Mycobacterium* genus-specific assay described below.

### 3.4. Aerosol Sampling

#### 3.4.1. Collection

Aerosol collection was carried out using a high-volume (low cut-off; 1100-1/min) impaction system (HVIS; Figure 2) linked to a 240 V voltage high performance vacuum pump [37,55]. The HVIS comprised a series of multistage round slit nozzle cascade impactors, which direct incoming air through four sterile polyurethane foam collection substrates labelled by position within the unit (top, upper and lower middle and bottom) [56,57] (Figure 2a,b). The HVIS was configured to collect particles in the ranges 10–2.5 µm and 2.5–0.1 µm, with the size being determined by the slit nozzles rather than the collection substrate [56] (Figure 2b). The HVIS was run for 3 h on the opposite river bank to the prevailing wind. Upon return to the laboratory, the multistage impactors were disassembled aseptically in a microbiological safety cabinet (Figure 2c), the circular foam collection substrates removed, cut into quarters using sterile scissors and placed at −80 °C in sterile plastic 50-mL centrifuge tubes (Figure 2d) until required. Each of the collection foams was processed separately. They were then aseptically transferred to stomacher bags and 20 mL sterile 1× PBS and placed in the stomacher (Stomacher^®^ 80 Biomaster; Seward Ltd., Worthing, UK). Stomaching was performed at the “high speed” setting (300 rpm ±5%) for 5 min at room temperature. The total volume of this suspension was then decanted into sterile 50 mL tubes and cells concentrated to 1 mL by centrifugation 16,000× g for 10 min at room temperature. This final suspension was then used for culture and DNA extraction for qPCR estimates of cell numbers of mycobacteria and *MAP*.

#### 3.4.2. Controlled Assessment of the Efficiency of Cell Recovery from Foam Substrates

To test the efficiency of recovery of cells foam substrates used in the HVIS aerosol collector, substrates were infused with *E. coli* JM101 (to give a representative sample of a fast growing general heterotrophy) and with *M. immunogenum* (a fast growing non-tuberculous mycobacterium, which aggregates into cell clumps, typical of mycobacteria). Foam substrates were cut into 1-cm strips and sterilised by autoclaving prior to use. Sterile foams were then placed into sterile 1.5-mL Eppendorf tubes and infused with 100 µL of cell suspension. This volume had previously been shown to be fully retained by the foam (not shown). Cultures of *E. coli* JM101 and *M. immunogenum* were grown to approximate cell concentrations of 10^8^ mL^−1^ before centrifugation for 3 minutes at 3000× g and re-suspension in sterile 1 ×PBS. Separate cell suspensions of each culture were serially diluted to 10^−7^ in 1× PBS to provide an estimate of how the efficiency of recovery changed across a range of concentrations. Infused foams were undisturbed for 1 h at room temperature. They were then aseptically transferred to stomacher bags and processed as described previously. The final suspension was then used for culture and DNA extraction for qPCR estimates of cell numbers. *E. coli* and *M. immunogenum* were cultured as above, and the efficiency of cell recovery was determined by comparing the colony forming units CFU mL^−1^ (or cell equivalents, see below) recovered to that of the original cultures used for infusion. Due to cell clumping that affects mycobacterial CFU counts, *M. immunogenum* cell equivalents [55] were also assessed by qPCR, as described below.

### 3.5. Direct Microscope Counts

Direct counts were carried out using DAPI staining [58] using a Leitz labarlux S epifluorescence microscope at ×1250 magnification under UV excitation at 358 nm and emission at 430 nm.

### 3.6. DNA Extraction

Total nucleic acids were extracted from all samples using the MagMax total nucleic acid extraction kit (P/N AM1840, Life Technologies, Paisley, UK) according to manufacturer’s instructions, apart from replacement of kit-supplied carrier nucleic acid with molecular reagent water (Sigma Aldrich Ltd., Poole, UK). Cell lysis by bead beating was performed on a FastPrep^®^-24 instrument (MP Biomedicals, Loughborough, UK) for 2 cycles of 1 min at a speed of 6.5 m/s (with 5 min at room temperature in between cycles). All samples were processed in sterile 1.5-mL microcentrifuge tubes. Nucleic acid extractions were also performed with 200 μL of PCR water (Sigma Aldrich Ltd., Poole, UK) as a negative control. DNA quality and concentration were determined using a Nanodrop 8000 spectrophotometer (Labtech International Ltd., Ukfield, UK). Finally, all total nucleic acids were stored at −80 °C.

### 3.7. PCR Amplification and qPCR

End-point PCR amplification was carried out using a Veriti thermal cycler (P/N 4375786, Life Technologies) in MicroAmp 96-well thin-walled reaction plates (P/N 4346906, Life Technologies). Amplification of the 16S *rrn* gene was carried out using primers pE and pH’ [59]; Table 4). Reactions (20 µL) were performed using and in strict accordance with the instructions provided with the AmpliTaq Gold 360 Master Mix system (P/N 4398881, Life Technologies).

**Table 4:** PCR primers and hydrolysis probes used in this study. * Our designation as oligonucleotides were originally simply the forward and reverse primer Taqman probe when described in van Coppenraet *et al*. (2004).

Oligonucleotide	Sequence and Fluorophore/Quencher (5'→3')	Target Gene	Reference
pE (forward)	AAACTCAAAGGAATTGACGG	Eubacterial 16S *rrn* gene	[59]
pH’ (reverse)	AAGGAGGTGATCCAGCCGCA	Eubacterial 16S *rrn* gene	
MimmFP (forward)	TTGATGTGCAGACGGATTCC	*M. immunogenum rpoB*	[55]
MimmRP (reverse)	CAACCTCGCGCCAACG	*M. immunogenum rpoB*	
MimmTP (hydrolysis probe)	VIC-TTGAATGGTTGGTCGGCTCGCC-TAMRA	*M. immunogenum rpoB*	
gMycFP * (forward)	GGGGTGTGGTGTTTGAG	*Mycobacterium* genus 16S-23S *rrn* gene ITS	[60]
gMycRP * (reverse)	CTCCCACGTCCTTCATC	*Mycobacterium* genus 16S-23S *rrn* gene ITS	
gMycP * (hydrolysis probe)	6FAM-TGGATAGTGGTTGCGAGCATC-TAMRA	*Mycobacterium* genus 16S-23S *rrn* gene ITS	
IS900qPCRF (forward)	GATGGCCGAAGGAGATTG	*M. avium* subsp. p*aratuberculosis* IS*900*	[61]
IS900qPCRR (reverse)	CACAACCACCTCCGTAACC	*M. avium* subsp. *paratuberculosis* IS*900*	
IS900qPCRTM (hydrolysis probe)	6FAM–ATTGGATCGCTGTGTAAGGACACGT–BHQ	*M. avium* subsp. *paratuberculosis* IS*900*	
F57-F (forward)	TACGAGCACGCAGGCATTC	*M. avium* subsp. *paratuberculosisF57*	[62]
F57-R (reverse)	CGGTCCAGTTCGCTGTCAT	*M. avium* subsp. p*aratuberculosisF57*	
F57 Taqman_mgb _(hydrolysis probe)	VIC-CCTGACCACCCTTC-MGB	*M. avium* subsp. *paratuberculosisF57*	

Where practicable, all real-time quantitative PCR amplifications were performed and reported in accordance with the MIQE guidelines [62]. Amplifications were carried out in a 7500 FAST Real-Time PCR system (Life Technologies, Paisley, UK) in MicroAmp optical reaction plates (P/N N801–0560, Life Technologies). Primers and hydrolysis probes specific to each of *Mycobacterium immunogenum*, *Mycobacterium* spp. and *Map* are listed in Supplementary Table 1.

*Map* was detected by qPCR using single-tube duplex reactions that combined the previously described assays [61,62] to amplify *Map*-specific regions IS*900* and F57, respectively. These assays comprised primers and hydrolysis probes described in Supplementary Table 1. Primer and probe concentrations were optimized such that each reaction (20 µL) contained the following: 10 µL of 2 × Environmental Master Mix (P/N 4396838, Life Technologies, UK); 1 µL (300 nM) of each primer (P/N 4304972, Life Technologies, UK) and 1 µL (250 nM) of each probe (P/N 450003, Life Technologies, UK); 2 µL of sterile PCR grade water (Sigma-Aldrich, UK); and 2 µL DNA. No template controls received sterilized PCR-grade water instead of DNA. The following cycling profile was used: one cycle of 95 °C for 10 min (for the activation of the AmpliTaq Gold enzyme) and 45 cycles of 95 °C for 15 s and 60 °C for 1 min. DNA used in standard curves was quantified and standard curves constructed in accordance with methods outlined in Rhodes *et al*., (2008), based upon a genome size of 4.83 Mbp for *M. avium* ssp. *paratuberculosis* K-10 (GenBank Accession No. NC002944) and an average number of gene copies per genome of 17 (IS*900*) and 1 (F57) [63,64]. The sensitivity of the IS*900* and F57 assays was determined for the present study using serially-diluted *Map* K-10 DNA. The limit of detection of the combined duplex assay of IS*900* and F57 was 20–50 cell equivalents (CE; see [55]). All reactions were carried out in triplicate. Inhibition of the PCR reaction was tested in a separate round of reactions after the initial MAP assessments by the addition of 2 ng of *Map* K-10 DNA (approximately 4 × 10^5^ genomes) to all samples and to control reactions where no sample DNA was added. No amplification, or a shift to a higher quantification cycle (Cq) value [65] when compared with the control reactions, was interpreted as inhibition.

## 4. Conclusions

Both river aerosol and shower unit results presented here are consistent with inhalation as a probable exposure route of *M. avium* subsp. *paratuberculosis* and other non-*Map* mycobacterial infections. Inhalation has been shown as a route for the infection of cattle [49,66]. *M. avium* subsp. *avium* demonstrates selective binding to pulmonary surfactant proteins [67]. Lung involvement is well described in adults with Crohn’s disease [67,68], and the disease in children often begins with a cough and a mild granulomatous tracheobronchitis [69,70,71,72,73]. Initial invasion via the oral route followed by *Map*’s substantial tissue tropism for the gut may result in chronic inflammation of the intestine [74,75]. *Although Map* is difficult to detect and even more difficult to culture, recent data has shown it to be significantly associated with Crohn’s disease and, if appropriate culture and PCR tests are done correctly, that almost everyone with chronic inflammation of the gut of the Crohn’s disease type is found to be infected with this chronic enteric pathogen [8,10,76,77,78,79,80]. Delivery of *Map* from showers and river aerosols may provide two previously undescribed human exposure routes for the pathogen significantly associated with Crohn’s disease.

## Supplementary Files

Supplementary File 1
