# Supplementary material for: Mycobacterium avium Subspecies paratuberculosis: Human Exposure through Environmental and Domestic Aerosols
Source: Pathogens. 2014 Jul 16;3(3):577–95. doi: 10.3390/pathogens3030577 (PMC4243430; doi:10.3390/pathogens3030577)
Supplement: Supplementary File 1 [file pathogens-03-00577-s001.pdf]

Supplementary

Table S1. PCR primers and hydrolysis probes used in this study. †

Oligonucleotide	Sequence and Fluorophore/Quencher (5′→3′)	Target Gene	Reference
pE (forward)	AAACTCAAAGGAATTGACGG	Eubacterial 16S *rrn* gene	Edwards *et al*. (1989)
pH’ (reverse)	AAGGAGGTGATCCAGCCGCA	Eubacterial 16S *rrn* gene	
MimmFP (forward)	TTGATGTGCAGACGGATTCC	*M. immunogenum rpoB*	Rhodes *et al*. (2008)
MimmRP (reverse)	CAACCTCGCGCCAACG	*M. immunogenum rpoB*	
MimmTP (hydrolysis probe)	VIC-TTGAATGGTTGGTCGGCTCGCC-TAMRA	*M. immunogenum rpoB*	
gMycFP* (forward)	GGGGTGTGGTGTTTGAG	*Mycobacterium* genus 16S-23S *rrn* gene ITS	van Coppenraet *et al*. (2004)
gMycRP* (reverse)	CTCCCACGTCCTTCATC	*Mycobacterium* genus 16S-23S *rrn* gene ITS	
gMycP* (hydrolysis probe)	6FAM-TGGATAGTGGTTGCGAGCATC-TAMRA	*Mycobacterium* genus 16S-23S *rrn* gene ITS	
IS900qPCRF (forward)	GATGGCCGAAGGAGATTG	*M. avium* subsp. p*aratuberculosis* IS*900*	Slana *et al.* (2008)
IS900qPCRR (reverse)	CACAACCACCTCCGTAACC	*M. avium* subsp. p*aratuberculosis* IS*900*	
IS900qPCRTM (hydrolysis probe)	6FAM–ATTGGATCGCTGTGTAAGGACACGT–BHQ	*M. avium* subsp. p*aratuberculosis* IS*900*	
F57-F (forward)	TACGAGCACGCAGGCATTC	*M. avium* subsp. p*aratuberculosis F57*	Schönenbrücher *et al*. (2008)
F57-R (reverse)	CGGTCCAGTTCGCTGTCAT	*M. avium* subsp. p*aratuberculosis F57*	
F57 Taqman$_{mgb}$ (hydrolysis probe)	VIC-CCTGACCACCCTTC-MGB	*M. avium* subsp. p*aratuberculosis F57*	

* Our designation as oligonucleotides were originally simply forward and reverse primers Taqman probe when described in van Coppenraet et al. (2004).

† This table is the same with Table 4 in text. The duplication caused by an omission.

© 2014 by the authors; licensee MDPI, Basel, Switzerland. This article is an open access article distributed under the terms and conditions of the Creative Commons Attribution license (http://creativecommons.org/licenses/by/3.0/).
